# Development of inCVAX, *In situ* Cancer Vaccine, and Its Immune Response in Mice with Hepatocellular Cancer

**DOI:** 10.4172/2155-9899.1000438

**Published:** 2016-07-18

**Authors:** Xiaoqiang Qi, Samuel SK Lam, Dai Liu, Dae Young Kim, Lixin Ma, Lu Alleruzzo, Wei Chen, Tomas Hode, Carolyn J Henry, Jussuf Kaifi, Eric T Kimchi, Guangfu Li, Kevin F Staveley-O’Carroll

**Affiliations:** 1 Department of Surgery, University of Missouri, Columbia, MO 65212, USA; 2 Ellis Fischel Cancer Center, University of Missouri, Columbia, MO 65212, USA; 3 Immunophotonics Inc., 4320 Forest Park Avenue #303, St. Louis, Missouri 63108, USA; 4 Veterinary Medical Diagnostic Laboratory, College of Veterinary Medicine, University of Missouri, Columbia, MO 65212, USA; 5 Department of Radiology, University of Missouri, Columbia, MO 65212; Harry S. Truman Memorial VA Hospital Biomolecular Imaging Center, USA; 6 Veterinary Medical and Surgery, College of Veterinary Medicine, University of Missouri, Columbia, MO 65212, USA; 7 Department of Molecular Microbiology and Immunology, University of Missouri, Columbia, MO 65212, USA; 8 Department of Molecular Microbiology and Immunology, University of Missouri, Columbia, MO 65212, USA

**Keywords:** Laser immunotherapy (LIT), *in situ* cancer vaccine (inCVAX), Hepatocellular cancer (HCC), Cytokine, T cell, Macrophage

## Abstract

Manipulation of immune system toward the rejection of established cancers has become the standard of care in some patients. Here we propose the development of an *in situ* autologous cancer vaccine, inCVAX, for the treatment of hepatocellular cancer (HCC). inCVAX is based on the induction of local immunogenic cancer cell death combined with local dendritic cell stimulation by intratumoral injection of the immune-activator *N*-dihydro-galacto-chitosan (GC). In a first set of experiments, cellular and molecular studies were performed to investigate the effect of inCVAX on immune activation in a murine model of HCC that we previously developed. Once large tumors were formed in mice, the tumor is surgically exposed and a laser fiber was inserted into the center of an individual tumor mass. Using a 10 mm diffuser tip, laser irradiation of 1.5 W was applied to heat the tumor at different durations (6-10 min) to assess tolerability of photothermal application at different temperatures. The laser application was followed by immediate injection of GC, and each mouse received one laser treatment and one GC injection. ELISA was used to assess the level of cytokines; immunohistochemical staining was conducted to analyze the effect of inCVAX on immune cell tumor-filtration and expression of tumor-specific antigens (TSAs) and tumor-associated antigens (TAAs). Results indicate that survival correlated to thermal exposure. At lower temperatures the photothermal effect was sufficient to induce tumor necrosis, but without obvious complication to the mice, although at these temperatures the treatment didn’t alter the level of TSAs and TAAs, so further optimization is suggested. Nevertheless, in response to the inCVAX treatment, cytotoxic cytokine IFN-γ was significantly increased, but suppressive cytokine TGF-β was dramatically reduced. Furthermore, inCVAX prompted tumor infiltration of CD3^+^, CD4^+^, and CD8^+^ T cells; but modulated macrophage subsets differently. In conclusion, while the protocol needs further optimization, it would appear that inCVAX for the treatment of HCC activates an immune response in tumor-bearing mice, which in turn may have potential for the treatment of HCC.

## Introduction

Hepatocellular cancer (HCC) is the most rapidly increasing type of cancer in the United States (US) [1] and accounts for 14,000 deaths per year [2]. HCC is refractory to traditional chemotherapies, and surgical resection or ablation offers a small chance for cure. Although liver transplant is an effective therapy in some cases, donor organs are scarce and often patients are not candidates for transplantation [3,4]. In 2008, the receptor tyrosine kinase inhibitor (RTKI), sorafenib, was approved by the Food and Drug Administration (FDA) to treat advanced HCC as it increased the median overall survival of patients from 7.9 to 10.7 months [5]. This small but statistically significant strategy reveals the challenging in treating this devastating disease. Development of the effective therapeutic strategies are urgently needed [4,6].

Immunotherapy represents one of the most promising approaches in the treatment of cancers [7]. For any cancer including HCC to progress it must evade the immune system; in fact, escaping immune destruction is now recognized a novel hallmark of cancer [8]. Priming antitumor immune response has therefore been validated to be a critical step in cancer immunotherapy [9]. In particular, the recent achievement of several key immunotherapy milestones dramatically changed the landscape of cancer treatment [10]. For example, the antibody-mediated blockade of cytotoxic T-lymphocyte antigen 4 (CTLA-4) and programmed cell death protein 1 (PD-1) were recently approved by the US FDA in the treatment of patients with advanced melanoma [11] and squamous non-small cell lung cancer [12]. Another approach that has shown promise is the application of therapeutic cancer vaccines, for example the recently approved TVEC [13], which has also been suggested to work synergistically with anti-PD-1 or other checkpoint inhibitors. These excited advances support the translation of immunotherapies to other cancers, but none are currently approved for the treatment of HCC.

Here we propose to explore inCVAX, an *in situ* autologous cancer vaccine (18) for HCC treatment, an approach that has been suggested to stimulate a robust antitumor immune reaction toward improving clinical outcomes [14]. InCVAX consists of two components: first, thermal treatment (for example with laser) of accessible tumors, releasing antigens and increasing immunogenicity; second, following thermal application, *N*-dihydro-galacto-chitosan (GC), a proprietary (Immunophotonics Inc.) potent immune-activator [15-17], is administrated into the heat-treated tumor site, enhancing the priming of immune responses [18]. As a result, combination of heat treatment and GC administration synergistically generate a potent immune response. Early studies have demonstrated that inCVAX destroyed both primary and metastatic tumors in some stage IV breast cancer patients, DMBA-4 metastatic mammary rat tumor model, feline acanthomatous ameloblastoma, and canine mast-cell tumors [19,20]. However, this strategy has not been tested in HCC due to absence of clinically relevant animal models. Recently we established a novel murine model of HCC which mimics the initiation and progression of human disease [21]. Using this model as a platform, we explore the application of inCVAX strategy in the treatment of HCC, and in a first set of experiments, we tested initial thermal parameters to make it suit for HCC treatment using interstitial laser as the heating source. In conjunction, preliminary effects of inCVAX on immune response in tumor-bearing mice were investigated. The current studies may advance the development of inCVAX in HCC treatment.

## Materials and Methods

### Mice

Six weeks old male C57BL/6 mice were purchased from the Jackson Laboratory (Bar Harbor, ME). Line MTD2 transgenic mice that express full-length SV40 Tag driven by the major urinary protein (MUP) promoter have been previously described [21]. All experiments with mice were performed under a protocol approved by the Institutional Animal Care and Use Committee (IACUC) in the Medical University of South Carolina. All mice received humane care according to the criteria outlined in the “Guide for the Care and Use of Laboratory Animals”.

### Antibodies

Antibodies for CD3 (Ab5690), CD4 (Ab183685), CD68 (Ab125212), alpha fetoprotein (α-AFP) (Ab46799), and Glypican-3 (GPC-3) (Ab66596) were purchased from Abcam Inc. (Cambridge, MA). Antibodies for SV40 TAg (v-300) (sc-20800) were purchased from Santa Cruz Biotechnology, Inc. (Dallas, TX). Antibodies for CD8a (14-0808) and F4/80 (14-4801-81) were purchased from Affymetric eBioscience, (San Diego, CA).

### Preparation of clinically relevant murine model of HCC

The preparation of a clinically relevant murine model of HCC was made as described [21,22]. Briefly, potential oncogenic hepatocytes were isolated from young male MTD2 mice. 0.5 × 10^5^ cells were seeded into the livers of male C57BL/6 mice by ISPL injection [21]. The androgen expression in recipient mice initiates the liver expression of oncogenic SV40 TAg under the control of liver specific promoter, driving tumor initiation and progression from the seeded hepatocytes.

### inCVAX of orthotopic HCC

Two major components constitute inCVAX: a thermal source and a proprietary immunoadjuvant. In this study an 805-nm diode laser (Diomedics, Woodlands, TX) was used as a thermal source. An optical fiber with diameter of 1.5 mm was connected to the laser with an SMA905 connector. The novel and proprietary immune-activator is *N*-dihydro-galacto-chitosan (GC) [15,18]. After animals were anesthetized with inhalational isoflurane, the abdominal cavity was opened to expose liver tumors. The laser fiber was inserted into the center of an individual tumor mass. Laser irradiation was conducted for 6 minutes, 8 minutes, and 10 minutes, which in turn was followed by injection of 100 μl GC. Each mouse only received one laser treatment and one GC injection.

### Magnetic resonance imaging (MRI)

Tumor surveillance was conducted with MRI. All MRI scans were obtained on a 7.0 T system (Bruker Biospin, Billerica, MA, USA) with in-plan resolution 0.1 mm and slice thickness 1 mm [21].

### Preparation and Hematoxylin & Eosin (H & E) staining of frozen tissue sections

As our previous performance [22], liver or tumor biopsies were freshly harvested from normal and tumor-bearing mice and frozen immediately by keeping tissue jar in a beaker of 2-methylbutane in dry ice. The frozen tissues were initially stored at −80°C, and transferred to −20°C on the day before sectioning on a cryostat (MICRON, HM 525, Thermo Scientific). The tissues were subjected to cryosectioning at a thickness of 10 μm and then placed on Superfrost plus slides (VWR International). Tissue sections were processed and stained with H&E as standard method. Briefly, the sections on Superfrost plus slides were stained as follows: wash in water, Richard Allan hematoxylin (VWR International) in aqueous solution for 60 s, wash in water, 1% acid alcohol for 1 dip, wash in water, 1% ammonia alcohol for 10 dips, wash in water, eosin (VWR International) for 10 s, followed by dehydration with 95%, 100% ethanol for 10 s, respectively, and xylene for 10 s.

### Immunohistochemistry (IHC)

Reagents used for IHC staining were obtained from Dako, (Carpinteria, CA). IHC to detect the expression of different molecules in tumors was performed as described [23]. Briefly, 5 μm formalinfixed paraffin-embedded tissue slides were dewaxed with xylene and rehydrated through ethanol/water dilutions. Antigen retrieval was performed using Dako Target Retrieval Solution (S1700, 1699) in a steamer at 95°C for 20 minutes, followed by 20 minutes at room temperature. The slides were kept in DAKO Wash Buffer (S3006) for 5 minutes. Endogenous peroxidases in the sections was quenched with DAKO ready-to-use dual-endogenous block solution (K4065), then washed with buffer. Sections were incubated with primary antibodies against CD3, CD4, CD8, F4/80, CD68, TAg, AFP and GPC-3 diluted in antibody diluent (S0809) for 90 min at room temperature. The primary antibodies were detected with corresponding Labelled Polymer-HRP, and then visualized by incubating the sections with DAB+ (DAKO, K3467) for 5-20 minutes at room temperature. Washes between incubations were carried out with TBS containing 0.05% Tween 20, pH 7.6 (DAKO, S1966). Sections were counterstained with hematoxylin (DAKO, S3302) for 1 min, then covered and mounted with mounting agent (Leica Surgipath MM 24, #3801120). The numbers of positive cells in each animal were counted in 3 randomly selected fields at 100 fold magnification by software ImageJ.

### Isolation of serum and enzyme-linked immunosorbent assay (ELISA)

Blood was drawn from the tumor-bearing mice with or without treatment, and the serum was isolated by brief centrifugation of the whole blood. The cytokine production in serum was measured with mouse Quantikine ELISA Kits specific for IFN-γ (Cat # DY485) and TGF-β1 (Cat # DY1679) from R&D systems according to the manufacture’s instruction. Optical density was measured by a microplate reader at 450 nm (Epoch Microplate Spectrophotometer, BioTek).

### Statistics

Paired data were analyzed using a 2-tailed paired Student’s t test. A P value of less than 0.05 was considered significant.

## Results

### Successful establishment of inCVAX treatment in HCC-bearing mice with large tumors

**Figure 1:**
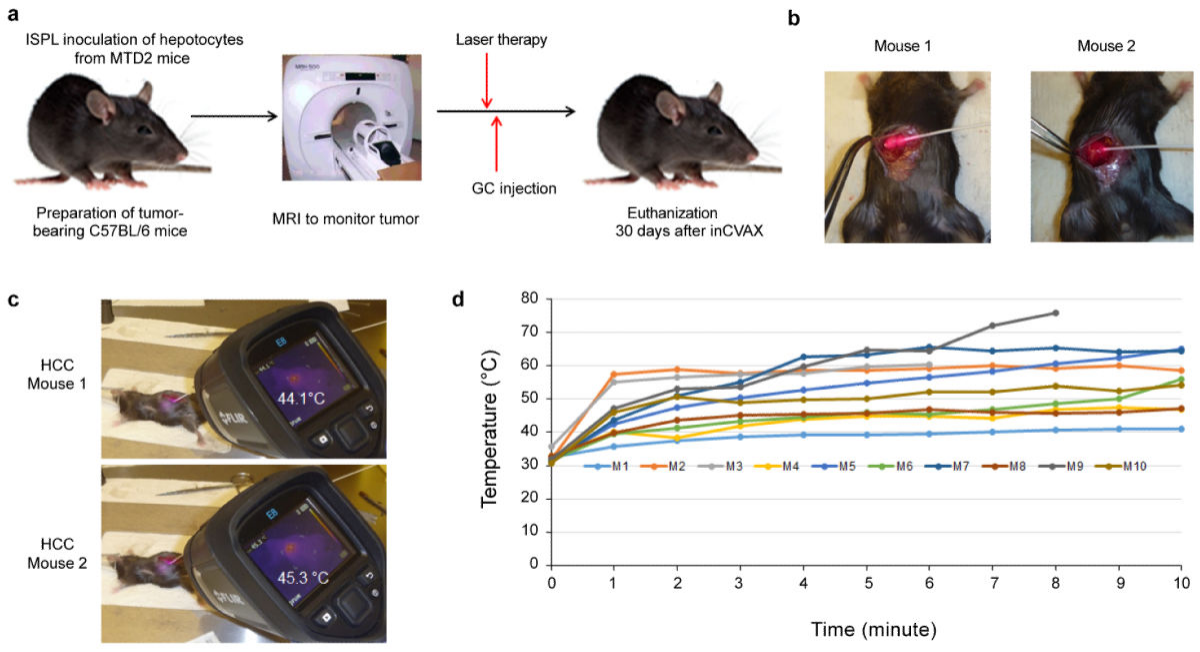
inCVAX of HCCs. **(a)** Schematic design of inCVAX treatment for HCC-bearing mice with large tumors. **(b)** Mice bearing with large tumors are anesthetized and the abdominal cavity was opened to exposure tumors. The fiber was inserted into the center of a tumor mass of about 8 mm in diameter. After performing laser treatment with the different parameters, 100 μl immunoadjuvant GC were injected into the tumors. **(C)** The temperature alteration over laser treatment was detected with a focus-free FLIR E8 Compact Thermal Imaging Camera. The representative temperatures generated with lase treatment in two tumor-bearing mice were showed. **(d)** The alteration of temperature over inCVAX in 10 tumor-bearing mice was recorded.

Using the established strategy developed previously by us, we made orthotopic HCC-bearing mice by seeding oncogenic hepatocytes from SV40 T antigen transgenic mice into livers of wild type mice by intrasplenic (ISPL) injection [21]. Tumor initiation and progression in recipient mice post ISPL injection were monitored by MRI. The established large tumor-bearing mice were used as a platform to explore inCVAX at different thermal parameters for the treatment of HCC (Figure 1a). To test tolerance for photothermal application in the liver, a laser fiber was inserted into the center of tumor to generate photothermal ablation of cancers (Figure 1b). The temperature alteration over laser treatment was detected with a focus-free FLIR E8 Compact Thermal Imaging Camera (Figure 1c and 1d). To complete the inCVAX treatment, an immune activator that consists of a semisynthetic functionalized glucosamine polymer, *N*-dihydro-galacto-chitosan (GC) [18] was injected into the center of the tumor following the thermal application. In total, 10 mice with large tumors were treated with different laser parameters. Two mice (M3 and M9) died during or immediately after thermal application due to bleeding following the insertion of the fiber. Five mice (M2, M5, M6, M7, M10) became very weak after fiber insertion and laser application when the temperature rose over 50°C, and they revived very slowly; therefore these mice were euthanized within one week as all of them showed sickness. Another three mice (M1, M4, M8) revived very fast after thermal treatment and survived very well when the temperatures stayed between 40°C and 50°C; no clinically relevant complications were found in these three mice within 30 days after receiving thermal application. The results suggested a correlation between temperature elevation in the liver, and survival of the treated mice.

**Figure 2:**
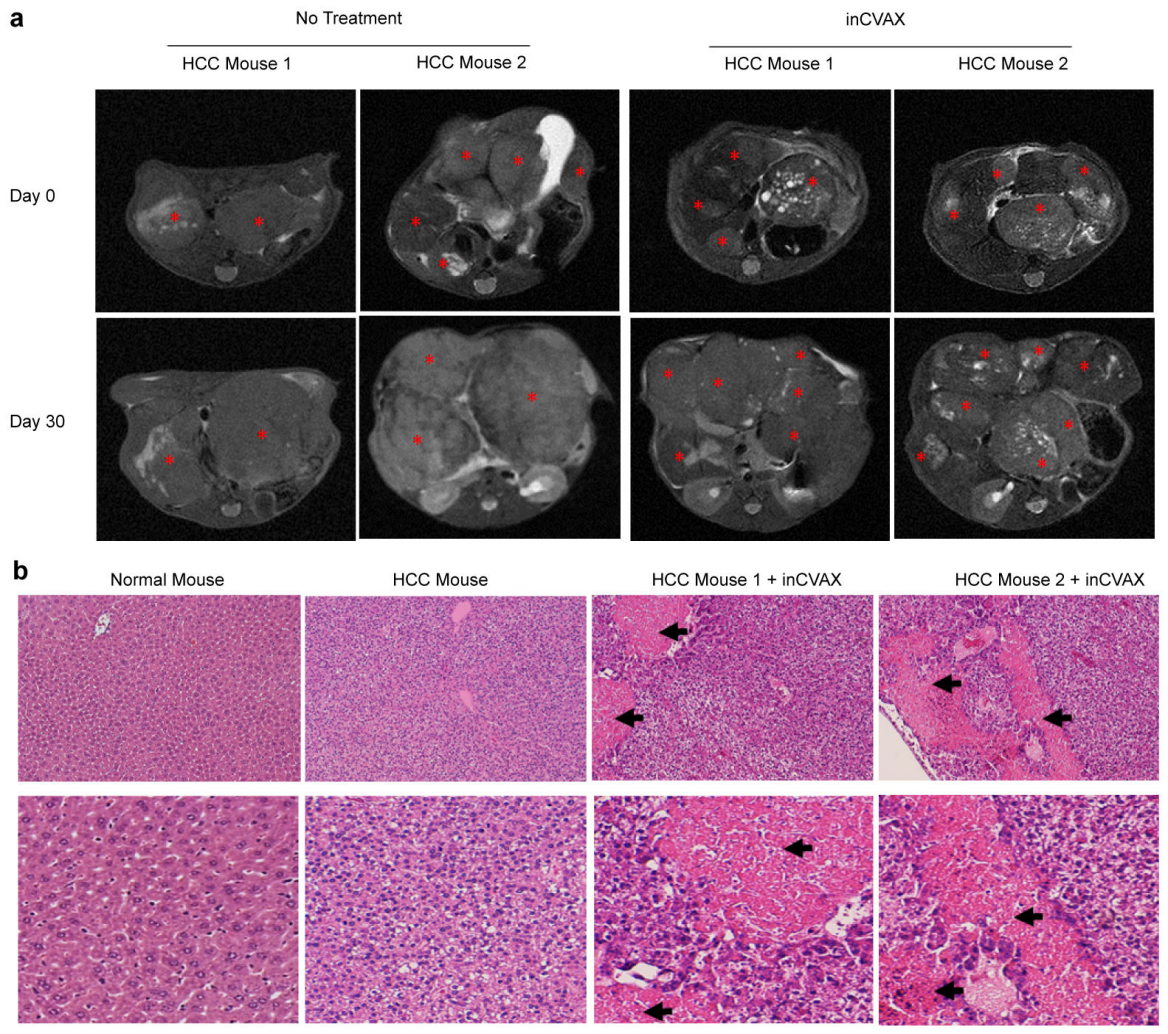
Representative MRI and H & E staining to show inCVAX-generated tumor damage. **(a)** MRI scan: Tumor mass in tumor-bearing mouse prior and post inCVAX treatment was monitored by MRI. Stars showed tumor mass. **(b)** H&E staining: Sections of liver tissues from normal mouse and tumor tissues in large tumor-bearing mice with and without inCVAX were stained. Arrows showed inCVAX-induced obvious tumor necrosis. Upper panel: low magnification; Lower panel: high magnification.

Thirty days post inCVAX treatment (i.e. thermal application combined with GC injection), MRI was performed in these three survival mice followed by immediate euthanization (Figure 2a). The tumor tissues were harvested and sectioned for H & E staining and the untreated tumor-bearing mice were used as controls (Figure 2b). Lower and high magnification of H & E staining demonstrated clear necrotic areas generated by inCVAX treatment which were unseen in untreated tumors. Under a microscope, coagulation necrosis was observed in the heated area, which had a clear boundary line with the surrounding tissue. These results suggested that laser treatment-mediated photothermal ablation with 1.5 watts for 10 minutes successfully efficiently kills tumor cells.

### inCVAX treatment modulates cytokine production in HCC-bearing mice

**Figure 3:**
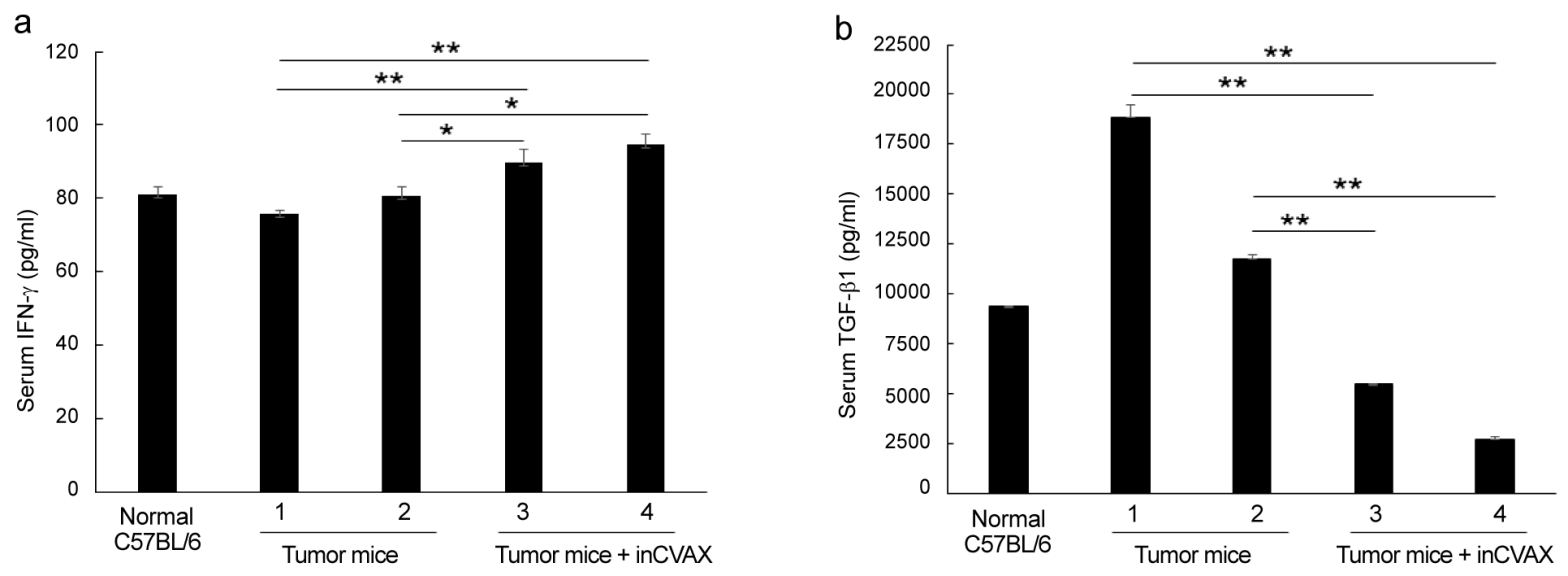
inCVAX modulates levels of serum cytokines in tumor-bearing mice. Blood was harvested from tumor-bearing mice with or without inCVAX and used to isolate serum. The level of cytokine IFN-γ **(a)** and TGF-β1 **(b)** were measured with ELISA in duplicate. n=2, ^*^p<0.05, ^**^ p<0.01, error bars represent mean ± SDs. inCVAX led to significant increase of IFN- γ, but reduction of TGF-β1 in large HCC-bearing mice.

To determine effect of inCVAX in immune response in HCC-bearing mice with large tumors, production of the cytotoxic cytokine IFN-γ [24] and well-documented suppressive cytokine TGF-β1 [25] was examined in the HCC-bearing mice in response to inCVAX treatment. Day 30 after inCVAX treatment, the blood was harvested to isolate serum. ELISA analysis detected about 90 pg/mL and 95 pg/mL of serum IFN-γ in two inCVAX-treated tumor-bearing mice (Figure 3a). The levels are significantly higher than untreated HCC-bearing mice (about 76 pg/mL in mouse 1 and 81 pg/mL in mouse 2). Conversely, inCVAX treatment led to the dramatic reduce in the level of TGF-β1 (5436 pg/mL and 2664 pg/mL) in comparison with untreated control tumor-bearing mice (18882 pg/mL and 11773 pg/mL) (Figure 3b). Significant increase of cytotoxic IFN-γ and dramatic reduce of suppressive TGF-β1 suggested that inCVAX treatment effectively activates immunity in HCC-bearing mice, possibly a Th1 response.

### inCVAX increases T cell infiltration into tumors

**Figure 4:**
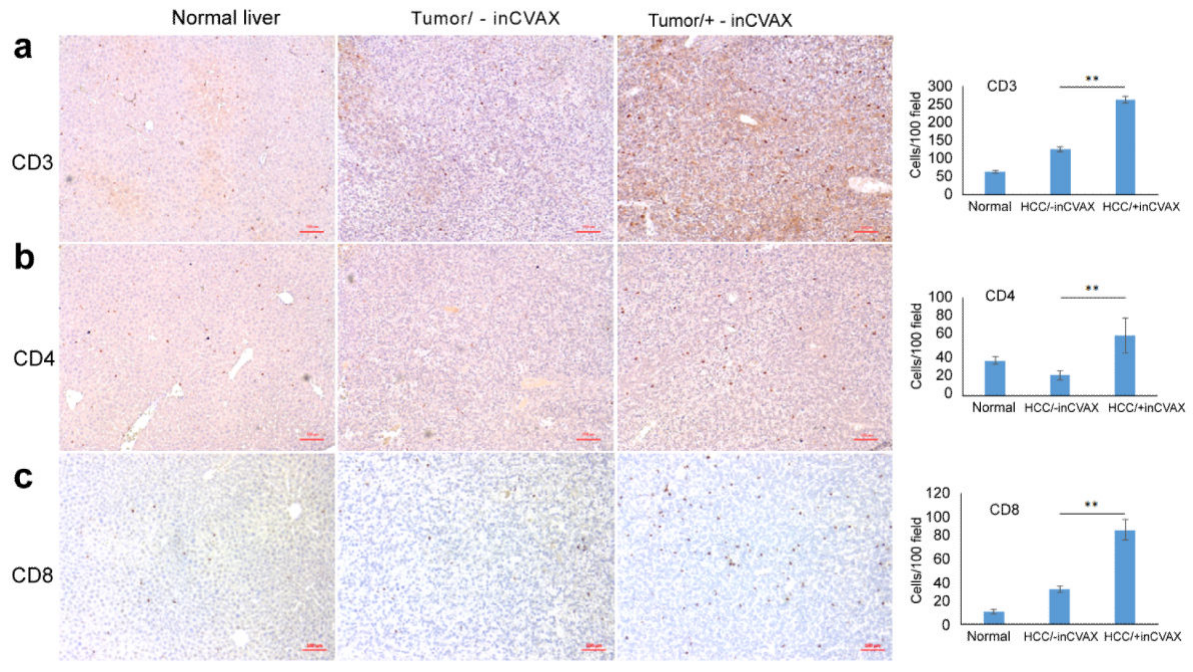
Effect of inCVAX on T cell infiltration into tumors. Immunohistochemical staining of CD3 **(a)**, CD4 **(b)**, and CD8 **(c)** was performed in normal liver tissues from control syngeneic wild type C57BL/6 mice and tumor tissues from large HCC-bearing mice with and without inCVAX. Few CD3^+^, CD4^+^, and CD8^+^ T cells were observed in normal mice and large HCC-bearing mice with equivalent level of CD4 and slight increase in HCC-bearing mice. In contrast, inCVAX led to high numbers of CD3^+^, CD4^+^, and CD8^+^ T cells detected in tumor tissues. The average liver- or tumor-infiltrated T cells per 100 fields were calculated. 100× magnification, n=3, ^*^p<0.05, ^**^ p<0.01, error bars represent mean ± SDs.

Generally, the presence of abundant T cells (particularly of the CD8^+^ subset) is associated with tumor regression and improved prognosis. Thus, the extent of T-cell infiltration was investigated in normal mice and tumor-bearing mice with or without inCVAX. Immunohistochemical staining showed very few detectable CD3^+^ T cells, CD4^+^ T cells, and CD8^+^ T cells in normal liver section from background matched C57BL/6 mice (left panel of Figures 4a-4c). Tumor growth resulted in the slight increase of CD3^+^ T cells and CD8^+^ T cells (middle panel of Figures 4a-4c). In contrast, the significant accumulation of CD3^+^ T cells, CD4^+^ T cells, and CD8^+^ T cells were detected in tumors from inCVAX-treated HCC-bearing mice (right panel of Figures 4a-4c). These results indicated that inCVAX extensively augmented T-cell infiltration into tumors.

### inCVAX differently modulates tumor infiltrating macrophage subtypes

**Figure 5:**
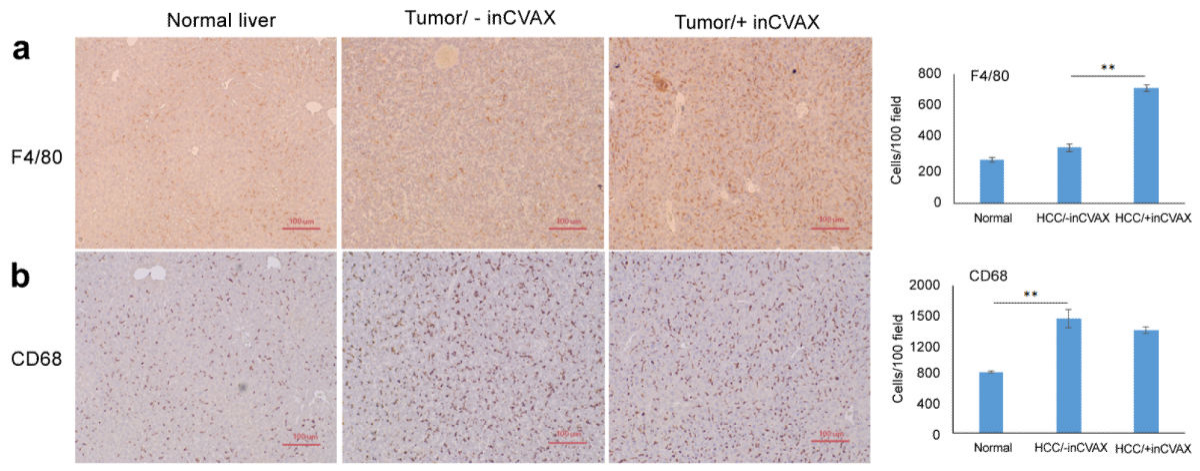
Effect of inCVAX on macrophage infiltration into tumors. Immunohistochemical staining of F4/80 **(a)** and CD68 **(b)** were performed in normal liver tissues from control syngeneic wild type C57BL/6 mice and tumor tissues from large HCC-bearing mice with and without inCVAX. Tumor progression led to slight increase of F4/80^+^ macrophages into tumor tissue. Additionally significant increase of tumor-infiltrating F4/80^+^ macrophages was detected in HCC-bearing mice received inCVAX. In contrast to F4/80^+^ macrophages, more CD68^+^ macrophages were stained in the liver of normal mice. Tumor growth dramatically drove more CD68^+^ macrophages infiltration into tumors. inCVAX slightly suppressed this trend. The average liver- or tumor-infiltrated T cells per 100 fields were calculated. 100 × magnification, n=3, ^*^p<0.05, ^**^p<0.01, error bars represent mean ± SDs.

Tumor-associated macrophages (TAMs) play a prominent role in the stromal and leukocyte compartment in malignancy, and also TAMs represent a cell type with an extreme functional plasticity enabling them to respond to different stimuli [26]. Here, typical CD68^+^ and F4/80^+^ macrophage subtypes and their tumor infiltration were assessed in HCC-bearing mice in response to inCVAX treatment (Figure 5). CD68^+^ and F4/80^+^ macrophages were detectable in normal liver from wild type C57BL/6 mice with more CD68^+^ macrophage population. Tumor progression significantly increased the tumor-infiltration of CD68^+^ macrophage (Figure 5b), but slightly enhanced F4/80^+^ macrophages (Figure 5a). InCVAX treatment led to dramatic increase of F4/80^+^ TAMs (Figure 5a), but slight reduced of CD68^+^ TAMs (Figure 5b). These results suggested that inCVAX differently impacted macrophage subtypes.

### Thermal effects on tumor-specific and tumor-associated antigens

**Figure 6:**
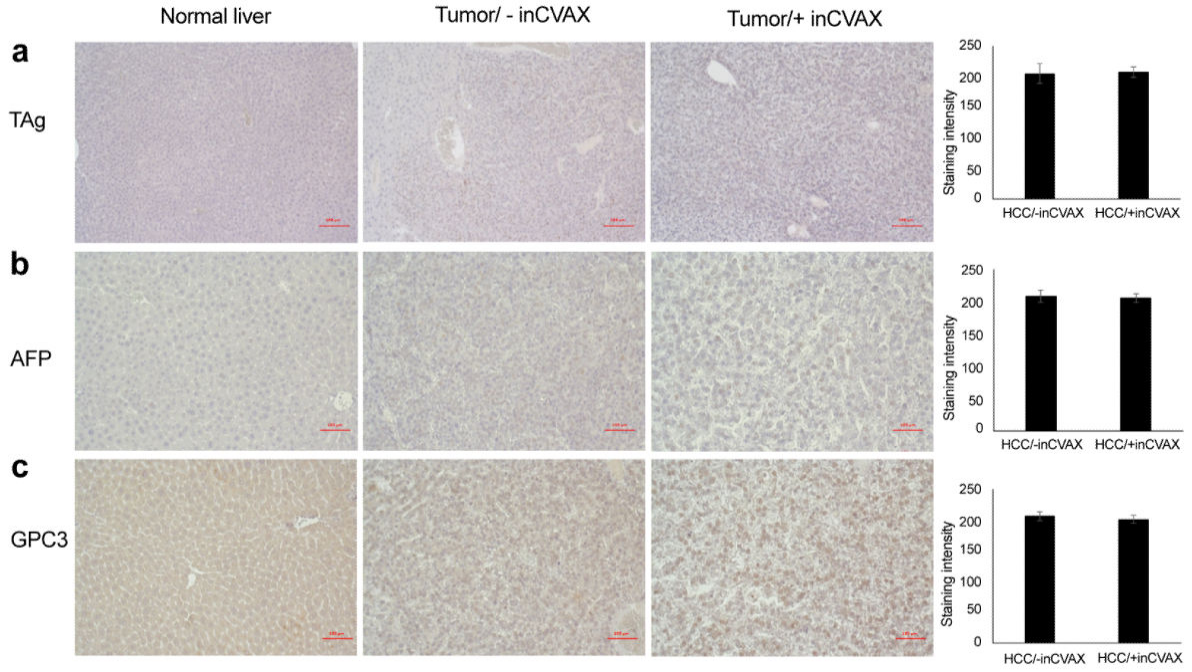
Influence of inCVAX on tumor antigens. Immunohistochemical staining of TAg **(a)**, GPC-3 **(b)**, and α-AFP **(c)** were performed in normal liver tissues from control syngeneic wild type C57BL/6 mice and tumor tissues from large HCC-bearing mice with and without inCVAX. In contrast to normal mice, specific expression of TAg and obviously increased production of GPC-3 and α-AFP were detected in tumor-bearing mice in photomicrographs at 100x magnification. There were no significant expression changes of three antigens detected in HCC-bearing mice after inCVAX treatment.

Tumor-specific antigens (TSAs) and tumor-associated antigens (TAAs) provide important targets for immunotherapy. In the current murine model of HCC, TAg serves as TSA due to their specific expression in tumors. In addition, AFP and GPC3 are HCC-related TAAs as both of them highly and frequently express in patients with HCC. We performed immunohistochemical staining of liver or tumors in the surviving mice (i.e. those that were exposed to the lower temperature range) to examine if thermal application modulates the level of TSA and TAA in HCC-bearing mice at temperatures between 40°C and 50°C. As shown in Figure 6a, no TAg was detected in normal mice, but highly expression was detected in the HCC-bearing mice, which was not affected by thermal treatment at this temperature range. Similarly, highly expression of AFP and GPC was found in HCC-bearing mice compared to normal mice; again, thermal application at temperatures between 40°C and 50°C didn’t led to alteration in the expression of both antigens. These results indicate that photothermal application at temperatures between 40°C and 50°C does not influence the expression of TSAs and TAAs. TSA and TAA expression was not investigated for temperature elevations above 50°C due to lack of survival of animals that were exposed to higher temperatures. Further work is required to optimize the thermal application in this tumor model [27].

## Discussion

In the current study, we explored different thermal parameters for the inCVAX-treatment of HCC. It was shown that laser irradiation is able to induce effective tumor necrosis in large tumor-bearing mice. At temperatures above 50°C, the thermal injury in the liver caused weakness of the animal, and the animals were sacrificed. At temperatures below 50°C, the treatment was well tolerated, but on the other hand, tumor-specific antigen (TSA)- and tumor-associated antigen (TAA)-expression was not modulated. Further optimization between thermal application and TSA- and TAA-expression is therefore suggested, either by using lasers at different wavelengths to induce a steeper thermal gradient in the tissue (i.e. lower transparency of the tissue, and thus less thermal heating of healthy tissue around the tumor), or by using other ablation techniques to liberate the desired tumor antigens.

In addition to exploring different thermal parameters, it was shown that immediate injection of the immune activator *N*-dihydro-galacto-chitosan (GC) following thermal application can activate immune response, which is implicated in prompting T cell tumor infiltration and differently modulating macrophage subsets.

Development of a successful inCVAX for large tumor treatment is an important contribution to HCC study. Laser ablation (LA) represents one of currently available loco-ablative techniques [28,29]. However, LA is only used in early HCC and has not been performed in large tumors. In addition, no effective approach capably overcomes the frequency tumor recurrence. Absence of clinically available animal model largely impedes the improved progression of LA treatment. Here we have used our murine model of HCC to perform initial optimization of laser irradiation parameters for clinically relevant treatment of large tumors. In the current study, we found that thermal application of laser led to the death of all treated mice once the induced temperature is over 50°C. In contrast, the treated mice are very tolerant to 40°C to 50°C and this temperature is high enough to induce tumor necrosis, albeit not to an alteration in the expression of TSAs and TAAs. It is possible, in the current experimental set-up, that the tissue transparency at the applied wavelengths, combined with the small absolute size of the tumors, caused too much surrounding liver tissue to heat up, hence inducing sickness to the animals at temperature elevation above 50°C. To induce higher temperatures in the tumor, while maintaining a lower temperature in the surrounding hepatic tissues, wavelenghts that display higher absorption in the tissue may be required. Alternatively, other ablation techniques may be used to reduce the thermal gradient. In summary, these results suggest that control of temperature is critical to perform successful laser ablation. In addition, orthotopic murine model plus the established inCVAX could provide an ideal platform towards improving HCC treatment in the future studies and advancing its clinical translation.

inCVAX causes the long-term immune activation which may lead to the more effective approach in the treatment of HCC. Tumor-induced profound immune suppression is a critical obstacle in the development of immunotherapy. Fortunately, it has been demonstrated that even after cancer develops, the power of the immune system can be harnessed to suppress tumor growth [30-32]. While experience with immunotherapy for HCC treatment is quite early, multifaceted approaches have shown efficacy in achieving disease regression and even cure [33]. Despite the preclinical and clinical studies have shown that laser treatment modulates the immune response, it is often too modest to exert potential effect against tumors. Comparing the untreated HCC-bearing mice, our cellular and molecular studies showed that significant increase of T-cell tumor infiltration, enhancement of cytotoxic IFN-γ, and reduction of suppressive TGF-β which could be detected in tumor-bearing mice 30 days post inCVAX treatment. In addition, we found that inCVAX differently regulates F4/80^+^ and CD68^+^ macrophages. Together, these results suggest that inCVAX strongly impact immune response in HCC-bearing mice with large tumors. Further studies are required to define the therapeutic efficacy on tumor regression and recurrence.

No effect of photothermal application on release of tumor antigens was detected at temperatures below 50°C. In our model, TAgs specifically express in tumors to serve as a TSA; highly expressed AFP and GPC3 as TAA which can be targeted to perform clinical relevant studies. By comparing levels of these three antigens by IHC at temperatures below 50°C, we demonstrated that moderate thermal application didn’t stimulate the release of TAg, AFP, and GPC3 into surrounding area as same level of these antigens were detected in large tumor-bearing mice with or without thermal application. Our finding is different from the report from another group where they found that laser-induced tumor cell death can release tumor antigens into the surrounding milieu [34], which can possibly be explained by difference in temperature exposure.

In conclusion, the inCVAX for the treatment of HCC is currently being developed. The successful inCVAX performance generates *in situ* tumor destruction which provides tumor-specific and tumor-associated antigens. Addition of immune-activator GC capably activate immune response in large tumor-bearing mice. Laser ablation in combination with immunoadjuvant injection may have potential for the treatment of HCC.

## Abbreviations

HCC: Hepatocellular Cancer
FDA: Food and Drug Administration
LA: Laser Ablation
ILT: Interstitial laser Thermotherapy
LIT: Laser Immunotherapy
CTLA-4: Cytotoxic T-lymphocyte Antigen 4
PD-1: Programmed cell death protein 1
inCVAX: *in situ* autologous cancer vaccine
GC: N-dihydro-galacto-chitosan
RTKI: Receptor Tyrosine Kinase Inhibitor
TAM: Tumor-associated Macrophage
TSA: Tumor-specific Antigen
TAA: Tumor-associated Antigen
